# Should Laparoscopic Surgery be an Outpatient Procedure?

**DOI:** 10.1155/1991/85742

**Published:** 1991

**Authors:** Karl A. Zucker

**Affiliations:** University of Maryland School of Medicine 22 South Greene Street Baltimore Maryland 21201 USA


4. Myburgh, J.A. (1990) Selective shunts: the Johannesburg experience. *Amer. J. Surg.*, **160**, 67–74
5. Maillard, J.N., Flamant, Y.M., Hay, J.M. and Chandler, J.G. (1979) Selectivity of the distal splenorenal shunt. *Surgery*, **86**, 663–670
6. Belghiti, J., Grenier, M.D., Nouel, O., Nahum, H. and Fekete, F. (1981) Long-term loss of Warren's shunt selectivity: Angiographic demonstration. *Arch. Surg.*, **116**, 1121–1124
7. Rikkers, L.F., Rudman, D., Galambos, J.T., Fulenwider, J.T., Millikan, W.J., Kutner, M., Smith, R.B., Salam, A.A., Jones, P.J. and Warren, W.D. (1978) A randomized controlled trial of the distal splenorenal shunt. *Ann. Surg.*, **188**, 271–282
8. Henderson, J.M., Millikan, W.J., Wright-Bacon, L., Kutner, M.H. and Warren, D.W. (1983) Hemodynamic differences between alcoholic and non-alcoholic cirrhosis following distal splenorenal shunt - Effect on Survival? *Ann. Surg.*, **198**, 325–334
9. Inokuchi, K., Beppu, K., Koyanagi, N., Nagamine, K., Hashizume, M. and Sugimachi, K. (1984) Exclusion of non-isolated splenic vein in distal splenorenal shunt for prevention of portal hypertension. *Ann. Surg.*, **200**, 711–717
10. Warren, D.W., Millikan, W.J., Henderson, K.M., Abu-Elmagd, K.M., Galloway, J.R., Shires, G.T., Richards, W.O., Salam, A.A. and Kutner, M.H. (1986) Splenopancreatic disconnection: Improved selectivity of distal splenorenal shunt. *Ann. Surg.*, **204**, 346–355
11. Henderson, J.M., Warren, W.D., Millikan, W.J., Galloway, J.R., Kawasaki, S. and Kutner, M.H. (1989) Distal splenorenal shunt with splenorenal disconnection: A 4 year assessment. *Ann. Surg.*, **210**, 223–341

J.A. Myburgh
Department of Surgery
University of the Witwatersrand
Medical School
York Road, Parktown
Johannesburg, 2193
South Africa

### SHOULD LAPAROSCOPIC SURGERY BE AN OUTPATIENT PROCEDURE?

### ABSTRACT

*Reddick, E.J. and Olsen, D.O. (1990) Outpatient laparoscopic laser cholecystectomy. The American Journal of Surgery,* **160**, 485-489.

**Laparoscopic laser cholecystectomy has been performed clinically in the United States since 1988. After refinement of the technique, the procedure was offered on an outpatient basis. Eighty-three patients underwent laparoscopic laser cholecystectomy during the study period. Thirty-seven (45%) had the procedure as an outpatient. Younger patients were more suited for the outpatient procedure and those without previous surgery were more likely to have the procedure done as an outpatient. Weight, operating time, and gallbladder pathology were similar, although patients with acute inflammation of the gallbladder were more likely to require hospitalization. The primary reason for patient admission was patient preference.**

**PAPER DISCUSSION**

KEY WORDS: Cholecystectomy, laparoscopic cholecystectomy, laparoscopic surgery, outpatient surgery

The immense popularity and rapid acceptance of laparoscopic cholecystectomy is without precedent in modern surgical history. Surgeons around the globe have eagerly rushed to the animal lab to acquire the necessary laparoscopic skills so that they may offer this procedure to their patients. This phenomenon has been largely brought about by consumer (i.e. patient) demand. Patients have been told through newspaper articles, television reports and word of mouth that laparoscopic surgery is far less painful, requires less time in the hospital and results in a more rapid return to their normal activities than conventional laparotomy and gallbladder removal. This article by Drs E.J. Reddick and D.O. Olsen is one of the few peer reviewed manuscripts which have thus far confirmed many of these early claims of laparoscopic cholecystectomy. The authors were among the first surgeons in the United States to perform laparoscopic biliary tract surgery and have one of the largest published clinical series to date[1]. In this manuscript Reddick and Olsen have demonstrated that in properly selected individuals laparoscopic cholecystectomy may be safely performed on an outpatient basis. This has become an important feature of laparoscopic surgery as health care cost containment has become a significant issue in the United States. Surgeons are now being encouraged to perform a variety of surgical procedures on an ambulatory basis which, only a few years ago, required two, three or more hospital days following surgery. The concept of performing cholecystectomy without hospital admission actually predates the development of laparoscopic biliary tract surgery. For many years surgeons have advocated performing cholecystectomy through smaller incisions. These clinical investigators have shown that by utilizing the so-called "mini-laparotomy" and avoiding the use of nasogastric tubes that one can minimize post-operative discomfort and ileus as well as shorten the in-hospital recovery interval[2,3]. This technique, however, has proven to be both technically challenging and difficult for most practicing surgeons to duplicate and therefore has not gained widespread acceptance. Instead it appears as if laparoscopic surgery will totally replace "mini-laparotomy" for cholecystectomy.

Although laparoscopic cholecystectomy is now widely practiced in many countries, the concept of performing this procedure on an outpatient basis has remained controversial. Concerns such as prolonged ileus, occult bleeding or intestinal injury, bile leak, post-operative pain, etc., have persuaded many surgeons to keep their patients in the hospital until the morning after surgery[4,5,6]. This report by Reddick and Olsen, however, clearly demonstrates that for properly selected patients, outpatient surgery is now a reasonable option. Although 45% of all patients were discharged the same day as laparoscopic cholecystectomy, the authors have not described in detail the criteria used for choosing appropriate patients. The best candidates appear to be those individuals who were younger, highly motivated, no history of prior abdominal surgery, operated upon electively for biliary colic, no evidence of choledocholithiasis and lived near the hospital. Another important feature not directly mentioned by Reddick is that the experience of the laparoscopic biliary tract surgeon is an important determinate in whether patients will be successfully managed as outpatients. In this series the average operating times were less than 90 minutes including the routine performance of intra-operative cholangiography. There were also no ductal injuries and the incidence of operative complications was very low. Therefore surgeons who are still mastering the necessary skills of laparoscopic surgery should not offer this option to their patients.

### REFERENCES

1. Spaw, A.T., Reddick, E.J. and Olsen, D.O. (1991) Laparoscopic Laser Cholecystectomy: An Analysis of 500 Procedures. *J. Surg. Lap. Endosc.*, **1**(1), 2–7
2. Dubois, F. and Berthelot, G. (1982) Cholecysteomie par Minilaparotomie. *Nouv. Presse. Med.*, **11**, 1139–1141
3. Ledet, W.P. (1990) Ambulatory Cholecystectomy Without Disability. *Arch. Surg.*, **125**, 1434–1435
4. Peters, J.H., Ellison, E.C., Innes, J.T., Liss, J.L., Nichols, K.E., Lomano, J.M., Roby, S.R., Front, M.E. and Carey, L.C. (1991) Safety and Efficacy of Laparoscopic Cholecystectomy: A Prospective Analysis of 100 patients. *Ann. Surg.*, **213**, 3–12
5. Schirmer, B.D., Edge, S.B., Dix, J., Hyser, M.J., Hanks, J.B., and Jones, R.S. (1991) Laparoscopic Cholecystectomy: Treatment of Choice for Symptomatic Cholelithiasis. *Ann. Surg.*, **213**(6), 665–677
6. Zucker, K.A., Bailey, R.W., Gadacz, T.R. and Imbembo, A.L. (1991) Laparoscopic Guided Cholecystectomy. *Am. J. Surg.*, **161**, 36–44

Karl A. Zucker
Associate Professor of Surgery
University of Maryland School of Medicine
22 South Greene Street
Baltimore, Maryland 21201
USA

